# Eosinophilic Colitis Presenting as Hemoperitoneum: A Report of a Rare Case

**DOI:** 10.7759/cureus.110676

**Published:** 2026-06-11

**Authors:** Jagadeesh Krishnamurthy, Akshay Krishnan

**Affiliations:** 1 Surgical Gastroenterology, Apollo Hospitals, New Delhi, IND; 2 Surgical Gastroenterology, Ramaiah Memorial Hospital, Bengaluru, IND

**Keywords:** emergency gastroenterology and endoscopy, eosinophilic gastritis, laparoscopic technique, spontaneous hemoperitoneum, use of steroids

## Abstract

Eosinophilic colitis (EC) is a rare clinical entity. Advanced cases with severe inflammation may develop severe abdominal pain and distension, malabsorption, intestinal obstruction, and even GI hemorrhage. Once diagnosed early, EC responds to steroid therapy. We report the case of a 46-year-old male who presented with acute-onset left upper abdominal pain, enlargement of the right scrotal sac, and hemoperitoneum. A contrast-enhanced CT scan of the abdomen revealed an irregular hypoechoic lesion measuring approximately 6.5 × 4.8 cm, likely a transverse mesocolon hematoma in the epigastric region with hemoperitoneum and right-sided hematocele with persistent processus vaginalis. USG-guided ascitic tap yielded bloody aspirate. Ascitic fluid analysis showed sheets of eosinophils (60%) with a hemorrhagic background. CBC showed eosinophilia (38%). He underwent diagnostic laparoscopy and laparoscopic left hemicolectomy; histopathological examination of the resected colon revealed EC (transmural type). He was given oral corticosteroids for two weeks after discharge. EC is a rare condition. It may present acutely and give rise to potentially severe and fatal complications like hemorrhage and perforation. With early diagnosis and appropriate treatment, we may be able to avoid serious complications in such patients.

## Introduction

Eosinophilic colitis (EC) is an uncommon inflammatory disorder within the spectrum of eosinophilic GI diseases, characterized by eosinophilic infiltration of the colonic wall [1]. Clinical manifestations are variable and include abdominal pain, diarrhea, weight loss, GI bleeding, intestinal obstruction, and, rarely, acute abdomen. Hemorrhagic presentations of EC are uncommon and have been limited to isolated reports of hemorrhagic diarrhea and ischemic colonic involvement [2]. Our review of the literature did not identify any previously reported cases of EC presenting with hemoperitoneum. We therefore report a rare case of EC presenting with spontaneous hemoperitoneum, demonstrating the importance of considering EC in patients with unexplained abdominal pathology and eosinophilia. This case highlights a potentially life-threatening manifestation, expanding the recognized clinical spectrum of this disease.

## Case presentation

A 46-year-old male with no comorbidities presented with acute-onset left upper abdominal pain of a colicky nature, lasting for two to three hours, with no radiation and partially relieved with IV analgesics. The patient also reported a history of loose stools, occurring five to six times daily over the preceding six months, for which he took symptomatic treatment from a nearby physician. There was no history of blood/mucus/pus in stools, fever, nausea, vomiting, rectal symptoms, loss of weight/appetite, or extraintestinal manifestations of IBD. There was an associated sudden increase in the size of the right hemiscrotum, with no other urological symptoms. There was no history of recent travel, outside food intake, or antibiotic use.

**Figure 1:**
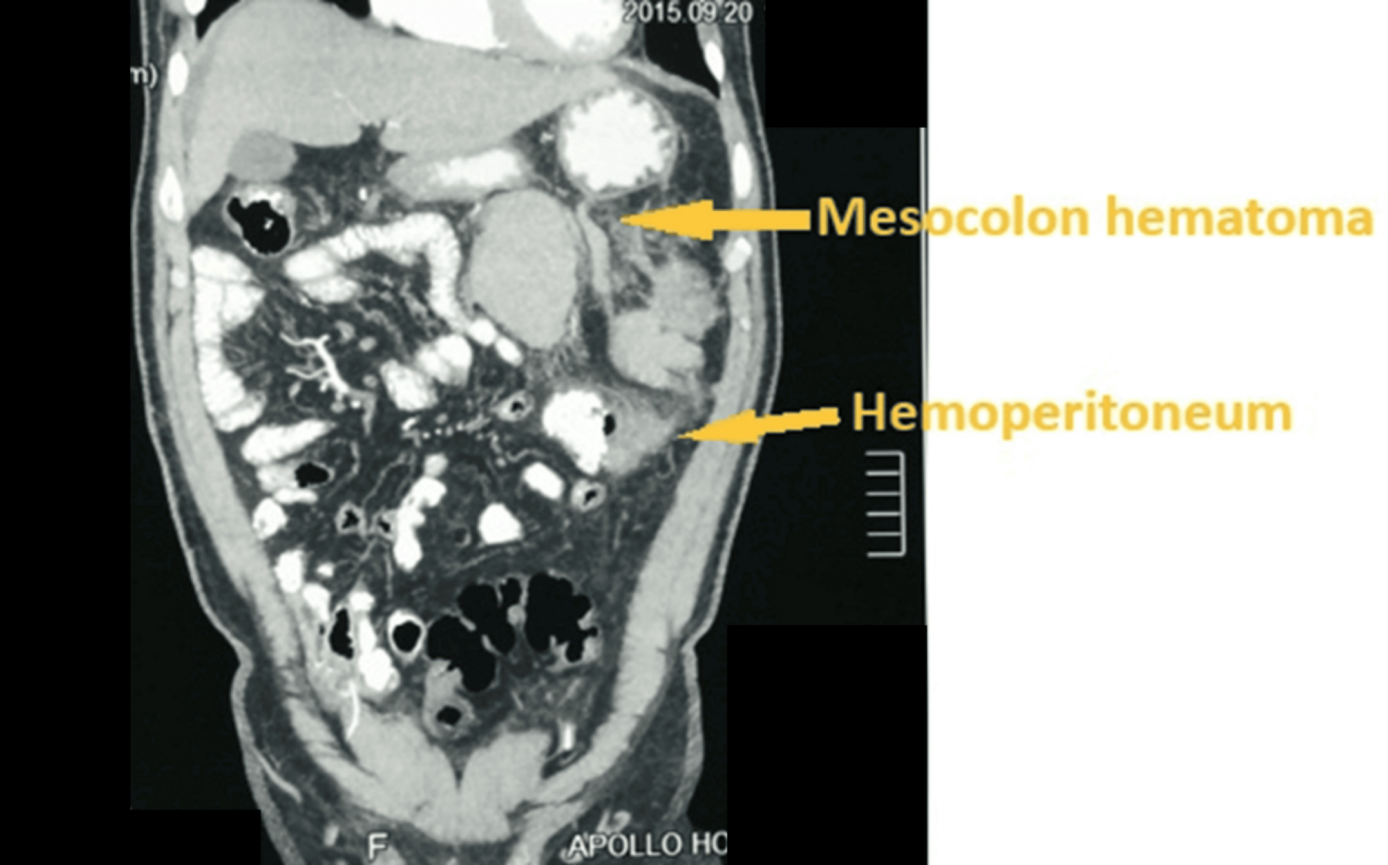
Coronal CT image showing mesocolon hematoma with hemoperitoneum

**Figure 2:**
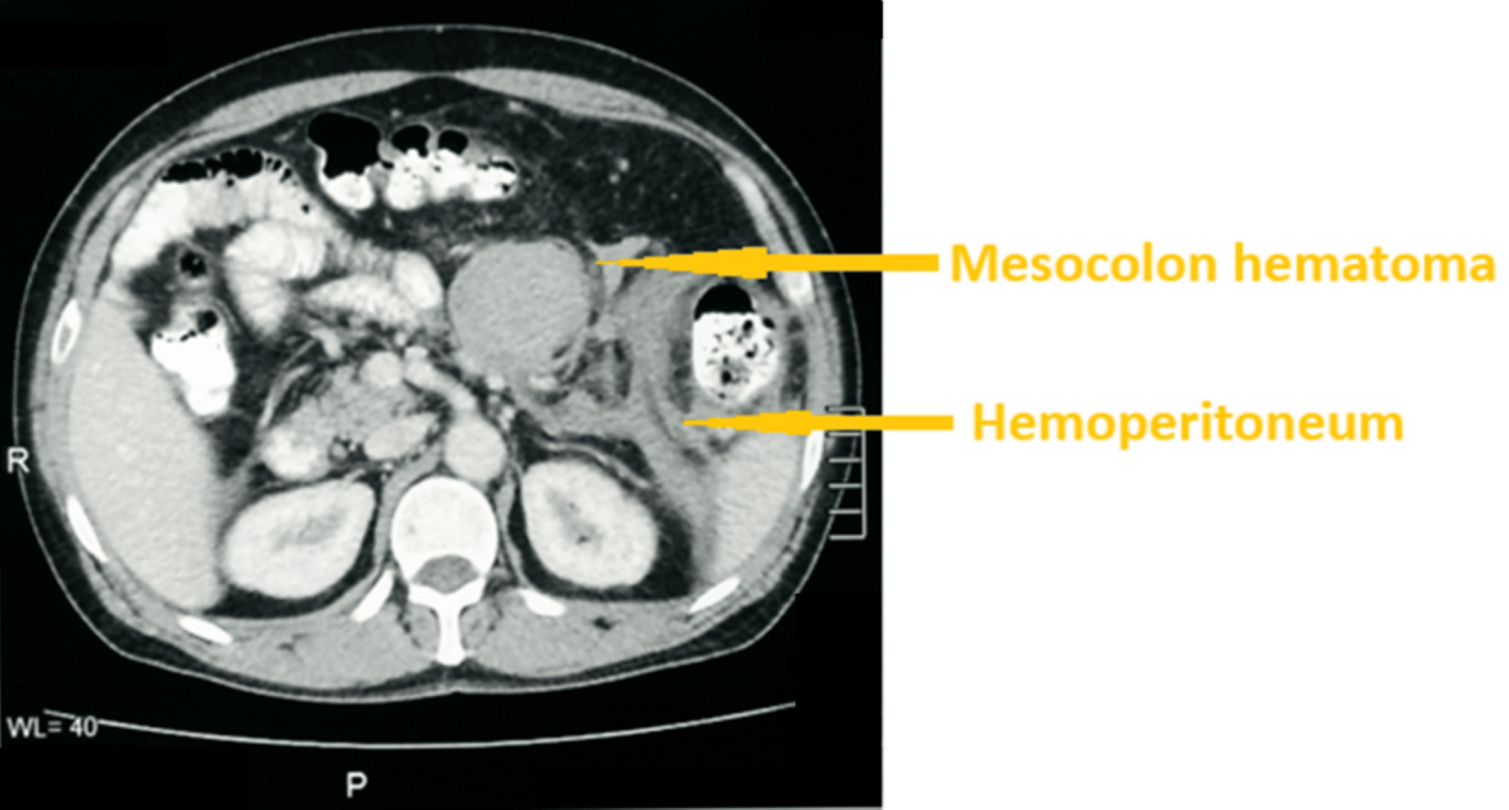
Axial CT image showing mesocolon hematoma with hemoperitoneum

On examination, he was hemodynamically stable and afebrile. Abdominal examination revealed tenderness in the left hypochondrium with a vague palpable mass measuring approximately 7 × 6 cm, mildly tender, mobile, and firm in consistency. A right-sided hematocele was noted, which was nontender. Contrast-enhanced CT of the abdomen demonstrated a hypoechoic lesion measuring approximately 6.5 × 4.8 cm (Figures 1, 2), suggestive of a transverse mesocolon hematoma with associated hemoperitoneum. A persistent processus vaginalis with a right-sided hematocele was also identified.

USG-guided ascitic fluid aspiration yielded hemorrhagic fluid. Cytological analysis revealed a predominance of eosinophils (approximately 60%). Peripheral blood examination showed marked eosinophilia (40%), while other routine blood tests, including CBC, erythrocyte sedimentation rate, CRP, kidney and liver function tests, and international normalized ratio, were normal, and stool analysis was unremarkable, with no ova/cysts.

**Figure 3:**
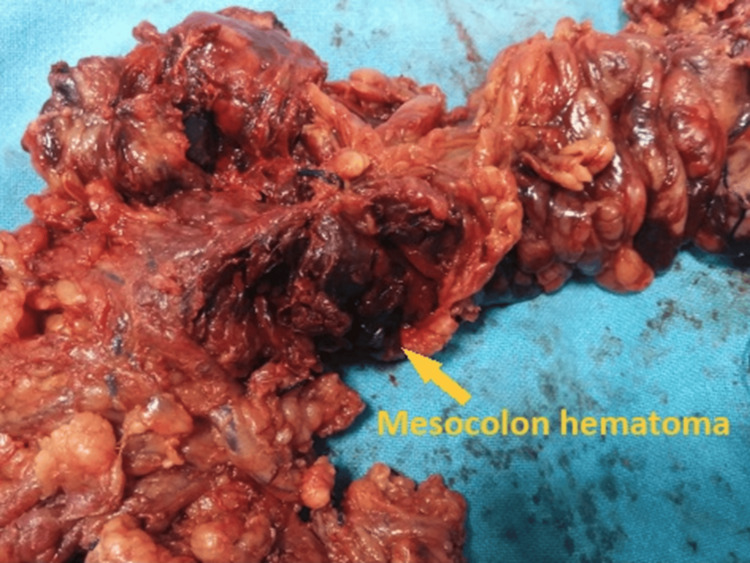
Intraoperative image showing mesocolon hematoma

**Figure 4:**
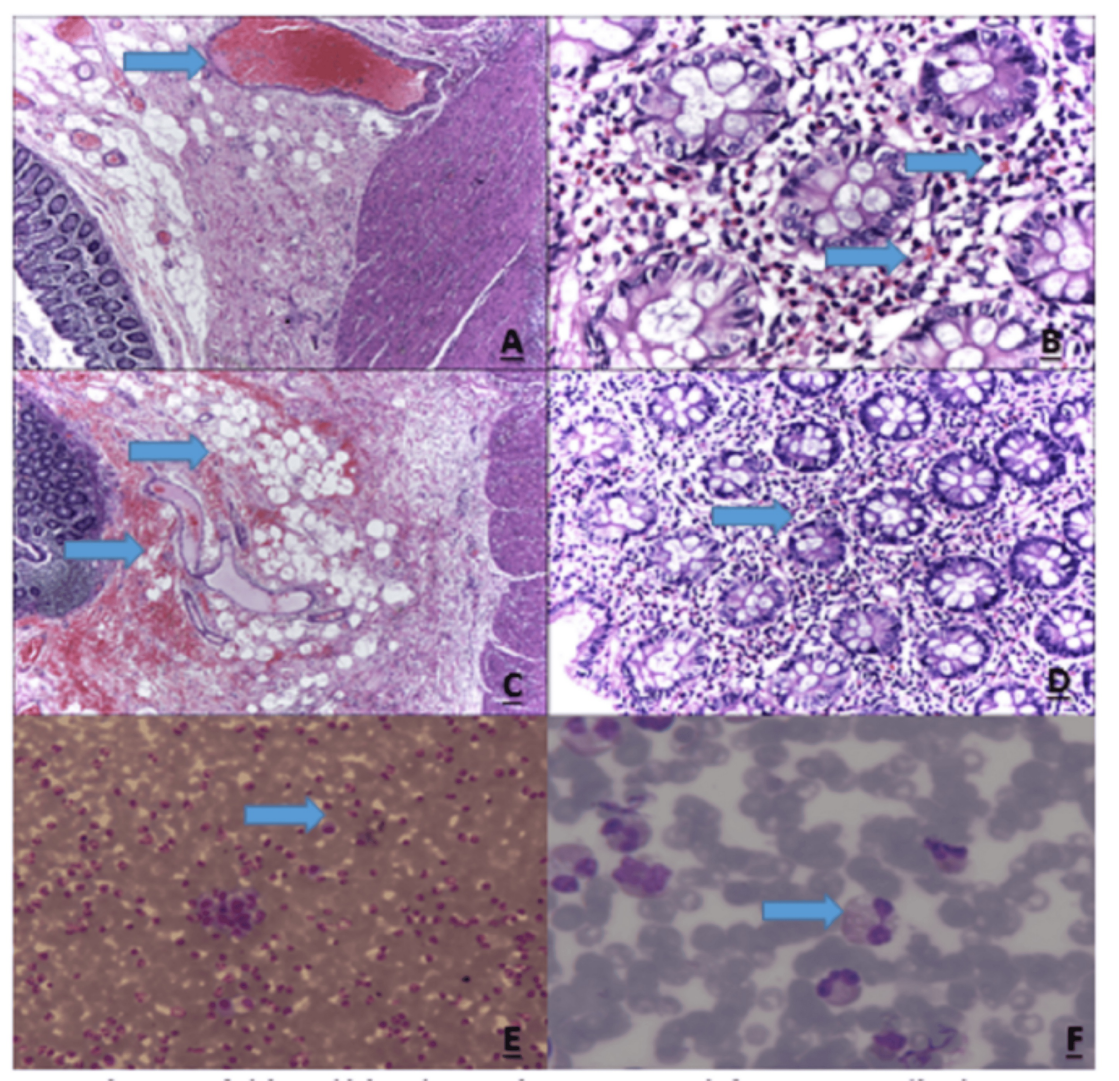
EC slides (A) Subserosal dilated blood vessels. (B) Eosinophils in mucosa (high-power view). (C) Submucosal hemorrhage showing RBCs. (D) Eosinophils in mucosa (low-power view). (E) Numerous eosinophils in ascitic fluid. (F) Eosinophils in ascitic fluid (high-power view). EC, eosinophilic colitis

With a preoperative diagnosis of hemoperitoneum and no etiological diagnosis, the patient was started on IV antibiotics and analgesics. In view of persistent pain, a diagnostic laparoscopy was performed, which revealed approximately 1 L of hemoperitoneum. A large hematoma was noted in the midportion of the transverse mesocolon, abutting the thickened, inflamed colonic wall. In view of these intraoperative findings, the procedure was converted to open surgery. Evacuation of the mesocolon hematoma (Figure 3), followed by left hemicolectomy with colocolic anastomosis, was performed. The postoperative period was uneventful. Histopathological examination confirmed transmural eosinophilic infiltration consistent with EC (Figure 4). The patient has been followed for 10 years and has been noted to be doing well, tolerating oral intake, with no episodes of loose stools. A recent complete hemogram showed an eosinophil count of 1%.

## Discussion

In the absence of secondary causes, eosinophilic infiltration of the GI tract characterizes a number of conditions identified as eosinophilic GI disorders. Among these, EC is the least common variant. Although the condition is more frequently described in the pediatric population, adult presentations are increasingly recognized.

The pathogenesis of EC remains poorly understood, with proposed mechanisms including immune dysregulation and hypersensitivity reactions. Primary EC is the rarest manifestation; the most common presentations are eosinophilic esophagitis and gastroenteritis [3]. The exact pathogenesis of EC remains unclear. Proposed mechanisms include immune dysregulation and hypersensitivity responses, resulting in eosinophilic recruitment and activation within the intestinal wall. Activated eosinophils release inflammatory mediators and cytotoxic proteins that can cause tissue damage and disruption of normal bowel architecture. In extensive disease, this inflammatory process may extend beyond the mucosa and involve deeper layers of the bowel wall. The hallmarks of EC are peripheral eosinophilia, segmental eosinophilic colonic infiltration, and functional abnormalities [4]. Secondary causes such as parasitic infections, drug exposure, inflammatory bowel disease, and systemic conditions must always be excluded before establishing a primary diagnosis.

The clinical manifestations of EC rely largely on the depth of bowel wall involvement. Mucosal disease typically presents with diarrhea and malabsorption [5], whereas transmural involvement may lead to bowel thickening, obstruction [6], or even perforation [7]. Serosal disease is often associated with eosinophilic ascites [8].

In the present case, the occurrence of hemoperitoneum represents an extremely unusual presentation. To the best of our knowledge, no previous cases of EC presenting with hemoperitoneum have been reported in the literature. We speculate that severe transmural eosinophilic infiltration may also affect mesenteric and subserosal vessels, leading to vascular injury, increased vascular permeability, and ultimately hemorrhage. This mechanism may explain the mesocolon hematoma and hemoperitoneum observed in our patient. The presence of mesocolon hematoma along with eosinophilic ascites suggests significant transmural involvement with vascular compromise, which likely contributed to hemorrhage.

The diagnosis of EC relied on a combination of clinical findings, peripheral eosinophilia, imaging, and histopathological confirmation [9], while carefully excluding secondary causes. In our patient, the marked eosinophilia in both peripheral blood and ascitic fluid, along with histopathological findings, supported the diagnosis.

Management strategies depend on the severity of presentation. While corticosteroids remain the mainstay of treatment in uncomplicated cases [10,11], surgical intervention becomes necessary in patients presenting with acute complications such as obstruction, perforation, or hemorrhage. In this case, the presence of hemoperitoneum necessitated urgent surgical management.

The case emphasizes how crucial it is to take EC into account when making a differential diagnosis for patients who have unexplained abdominal symptoms and eosinophilia, especially when imaging reveals atypical findings. Finally, although rare, EC can present with life-threatening hemorrhagic complications, including hemoperitoneum, thereby expanding the known clinical spectrum of this uncommon disorder.

## Conclusions

EC is a rare condition characterized by a broad spectrum of clinical presentations. It may occasionally present with acute and life-threatening complications such as hemoperitoneum. Early recognition and appropriate management are essential for preventing adverse outcomes. This case emphasizes the need for a high index of suspicion, particularly in patients with unexplained eosinophilia and atypical intra-abdominal findings.
